# Elevated inflammatory fecal immune factors in men who have sex with men with HIV associate with microbiome composition and gut barrier function

**DOI:** 10.3389/fimmu.2022.1072720

**Published:** 2022-12-20

**Authors:** Katherine M. Littlefield, Jennifer M. Schneider, Charles P. Neff, Victoria Soesanto, Janet C. Siebert, Nichole M. Nusbacher, Nancy Moreno-Huizar, Ian M. Cartwright, Abigail J. S. Armstrong, Sean P. Colgen, Catherine A. Lozupone, Brent E. Palmer

**Affiliations:** ^1^ Department of Medicine, University of Colorado, Aurora, CO, United States; ^2^ CytoAnalytics, Denver, CO, United States; ^3^ Department of Biomedical Informatics, University of Colorado Anschutz Medical Campus, Aurora, CO, United States

**Keywords:** human immunodeficiency virus (HIV), inflammation, men who have sex with men (MSM), gut microbiome, immune factors, cytokines

## Abstract

**Introduction:**

People living with HIV infection (PLWH) exhibit elevated levels of gastrointestinal inflammation. Potential causes of this inflammation include HIV infection and associated immune dysfunction, sexual behaviors among men who have sex with men (MSM) and gut microbiome composition.

**Methods:**

To better understand the etiology of gastrointestinal inflammation we examined levels of 28 fecal soluble immune factors (sIFs) and the fecal microbiome in well-defined cohorts of HIV seronegative MSM (MSM-SN), MSM with untreated HIV infection (MSM-HIV) and MSM with HIV on anti-retroviral treatment (MSMART). Additionally, fecal solutes from these participants were used to stimulate T-84 colonic epithelial cells to assess barrier function.

**Results:**

Both MSM cohorts with HIV had elevated levels of fecal calprotectin, a clinically relevant marker of GI inflammation, and nine inflammatory fecal sIFs (GM-CSF, ICAM-1, IL-1β, IL-12/23, IL-15, IL-16, TNF-β, VCAM-1, and VEGF). Interestingly, four sIFs (GM-CSF, ICAM-1, IL-7 and IL-12/23) were significantly elevated in MSM-SN compared to seronegative male non-MSM. Conversely, IL-22 and IL-13, cytokines beneficial to gut health, were decreased in all MSM with HIV and MSM-SN respectively. Importantly, all of these sIFs significantly correlated with calprotectin, suggesting they play a role in GI inflammation. Principal coordinate analysis revealed clustering of fecal sIFs by MSM status and significant associations with microbiome composition. Additionally, fecal solutes from participants in the MSM-HIV cohort significantly decreased colonic transcellular fluid transport *in vitro*, compared to non-MSM-SN, and this decrease associated with overall sIF composition and increased concentrations of eight inflammatory sIFs in participants with HIV. Lastly, elevated levels of plasma, sCD14 and sCD163, directly correlated with decreased transcellular transport and microbiome composition respectively, indicating that sIFs and the gut microbiome are associated with, and potentially contribute to, bacterial translocation.

**Conclusion:**

Taken together, these data demonstrate that inflammatory sIFs are elevated in MSM, regardless of HIV infection status, and are associated with the gut microbiome and intestinal barrier function.

## 1 Introduction

Pathogenesis of HIV infection is closely tied with the gastrointestinal (GI) tract because it is a major site of HIV replication. Large numbers of activated CCR5-expressing CD4^+^ T cells, which are specifically targeted by HIV, reside in the gastrointestinal tract (1). These cells fuel HIV infection in the gut, resulting in profound depletion of T cells in the lamina propria and chronic inflammation (2). Intestinal inflammation can promote the breakdown of the epithelial barrier and bacterial translocation, which in turn leads to systemic immune activation/inflammation (3) that contributes to HIV pathogenesis and disease progression (4). Furthermore, while gut inflammation and impaired barrier function improve with ART, these GI issues persist (5), and have been linked with metabolic (6), and other co-morbidities (7) in people living with HIV (PLWH) on ART. Microbiome differences, such as lower alpha diversity in untreated individuals with low CD4^+^ T cell counts (8) or ART-treated individuals with low NADIR (9), have been observed in PLWH (10). In addition, bacteria from fecal material of HIV-positive individuals has been shown to induce higher immune activation *in vitro* (11). Furthermore, others have shown that compositional shifts in the fecal microbiome associated with HIV correlated with changes in metabolic function and production of cytokines detectable in plasma samples (12, 13) and mucosal biopsies (14).

In the United States men who have sex with men (MSM) comprise over 60% of new cases of HIV infection annually (15). We and others have shown gut microbiome composition in MSM regardless of HIV infection is highly altered compared to seronegative non-MSM (10, 16), and is characterized by high relative abundance of the bacterial genus *Prevotella* and low *Bacteroides*, as well as many additional differentiating taxa (16, 17). Intestinal microbiome composition has also been associated with the risk of HIV acquisition in MSM (14). Whole fecal bacterial communities isolated from the stools of MSM with and without HIV induce immune activation and increase HIV infection of lamina propria mononuclear cells *in vitro (* 11). Additionally, gavage of fecal bacteria from these MSM cohorts into gnotobiotic mice leads to elevated levels of intestinal immune activation compared to non-MSM controls (18). This has been linked in part through particular enriched bacteria in MSM such as *Holdemanella (* 19). Because of the importance of gut immune activation for HIV pathogenesis, transmission among MSM, and co-morbidity, in-depth profiling of inflammation in the gut is essential for understanding these processes.

Measurement of soluble immune factors (sIF) present in feces, including chemokines, cytokines, growth factors and other signaling factors, is an attractive method for studying gut immune activation since collection of fecal material is relatively non-invasive. Although such methods have been used to assess GI inflammation in the context of GI diseases (20–23), how they differ with HIV infection, treatment, and MSM status has not been explored nor has whether their levels associate with intestinal microbiome composition or effect barrier function. Here we assessed well-vetted measures of intestinal inflammation, fecal sIFs, microbiome composition and markers of bacterial translocation to characterize and gain mechanistic insights into the relationship between intestinal inflammation and barrier function in HIV infection and in MSM, using stool samples collected from HIV-seronegative MSM (MSM-SN), MSM with HIV infection with (MSM-ART) and without ART treatment (MSM-HIV) and male non-MSM-SN participants. Taken together, our data demonstrate that inflammatory sIFs are elevated in MSM and with HIV infection, associate with gut microbiome composition, and negatively influence intestinal barrier function in HIV.

## 2 Materials and methods

### 2.1 Study participants

Participants from the Denver metropolitan area were recruited under study protocol #14-1595 approved by the Colorado Multiple Institutional Review Board (CoMIRB). All participants provided written consent prior to collection of data and samples and were separated into cohorts based on sex, HIV infection and current use of ART, and sexual behavior. All participants on ART underwent treatment for at least 12 consecutive months using a minimum of three separate ART medications prior to study entry and displayed at least six consecutive months of viral suppression. Participants not undergoing ART were either never treated or off treatment for six consecutive months prior to study participation. Use of antibiotics within three months of sample collection, diagnosis with an active gastrointestinal disease, opportunistic/chronic infection or malignancy, and/or prescription of anticoagulant or hypoglycemic medications were exclusionary for this study. The four cohorts of male participants were MSM with HIV, not currently using ART (MSM-HIV: n=15), HIV-positive MSM undergoing ART (MSM-ART: n=13) and HIV-seronegative men who either had sex with men (MSM-SN: n=17) or who did not (non-MSM-SN: n=14). A cohort of female participants with HIV and on ART treatment (F-HIV: n=5) were also included and compared to a matched cohort of women with no history of HIV infection (F-SN: n=5). Analysis of the female cohorts was limited due to the small cohort size. All cohort demographics data are included in **Table 1**.

**Table 1:** Participant cohort demographics and characteristics.

	Male				Female	
	Non-MSM-SN	MSM-SN	MSM-ART	MSM-HIV	F-SN	F-HIV
N	14	17	13	15	5	5
Race (BA/A/W/O)^a^	0/1/13/0	1/0/17/0	2/0/12/0	2/0/13/0	0/0/4/1	0/0/5/0
Ethnicity (H/NH)^b^	2/12	1/17	0/13	2/13	1/4	2/3
Median age (years)	32 (22-70)	35.5 (27-50)	56^c^ (44-65)	36 (23-55)	29 (24-61)	54 (30-63)
Median HIV-1 viral load (RNA copies/mL)	NA	NA	0 (0-20)	62800^d^ (159-5.2e5)	NA	20 (0-20)
Median CD4^+^ T cell count (cells/μL)	NA	NA	648 (177-1114)	577 (201-939)	NA	639 (187-1417)
Median fecal BCA (ug/mL)	881.7	829.8	700.1	763.8	868.6	1025.8

Kruskal-Wallis tests were performed for all demographic characteristics, corrected for multiple comparisons and significant differences are indicated. Units are provided in parentheses in the first column and all information in parentheses in subsequent columns are data ranges.

a BA, Black/African American; A, Asian; W, White; O, Other.

b H, Hispanic; NH, non-Hispanic.

c The median age of MSM-ART is significantly higher compared to all cohorts excluding F-HIV. (Non-MSM-SN: P = 0.0017; MSM-SN: P = 0.0060; MSM-HIV: P=0.0051; F-SN: P=0.020).

d P < 0.0001 in comparison to MSM-ART and P=0.0056 in comparison to F-HIV. NA, Not Applicable.

### 2.2 Collection of fecal samples and surveys

Participants collected full stool samples in a commode specimen collector prior to their clinic visit, which were shipped or transported within 48 hours either frozen or cool. Upon delivery, samples were transferred to long-term storage at -80°C. During research visits, participants completed a GI-symptoms questionnaire based on the GSRS (24) comprised of 14 multiple choice questions covering common GI issues experienced within the prior year and 24 hours before stool sample collection, including diarrhea, constipation, bloating, flatulence, vomiting and abdominal pain. Multiple choice answers ranged from “1 – little to no symptoms” to “4 – debilitating symptoms”. An aggregate GI Symptoms Score was calculated as the average value across all 14 questions. Participants were also asked to evaluate the consistency of their stool sample at the time of collection by utilizing the visual Bristol stool scale (25). Each participant assigned a subjective score (1-7) that most closely resembled their fecal sample.

### 2.3 Fecal solute preparation and sIF quantification

Fecal solute preparations were adapted from previously described methods with significant alterations (21, 22). Specifically, an aliquot of two grams of frozen feces was obtained and mixed with 8 mL of saline solution (DPBS, Protease Inhibitor, EDTA, DNase). Samples were homogenized for 1 min and placed on ice for 30 min. Samples were then ultra-centrifuged at 12,000 rpm for 45 min at 5°C, and the supernatant was passed through a 0.2 μm filter, aliquoted and stored at -80°C until testing. Total protein levels were assessed using a Bicinchoninic acid (BCA) assay. Standard sandwich ELISAs were used to measure fecal calprotectin (Epitope Diagnostics, San Diego, CA), IL-22 (eBioscience/Thermofisher, Waltham, MA), sCD14 (Hyclone, Uden, Netherlands) and sIgA (BioVendor, Brno, Czech Republic), while multi-plex ELISAs (MesoScale Diagnostics, Rockville, MD) were used to measure multiple analytes simultaneously each following manufacturer’s protocols. The following V-Plex MSD (MesoScale Diagnostics, Rockville, MD) kits were utilized: Proinflammatory Panel 1 (IFN-γ, IL-1β, IL-2, IL-4, IL-6, IL-8, IL-10, IL-12p70, IL-13, TNF-α), Vascular Injury Panel 2 (SAA, CRP, VCAM-1, ICAM-1), and Cytokine Panel 1 (GM-CSF, IL-1α, IL-5, IL-7, IL-12/23p40, IL-15, IL-16, IL-17A, TNF-β, VEGF-A).

### 2.4 DNA extraction and sequencing

DNA was extracted from the same fecal samples used in the ELISAs using the standard Power Soil Kit protocol (Qiagen). Extracted bacterial DNA was PCR amplified with barcoded primers targeting the V4 region of 16S rRNA according to the Earth Microbiome Project (EMP) standard protocols (http://www.earthmicrobiome.org). Each PCR product was quantified using PicoGreen (Invitrogen, Carlsbad, CA), and equal amounts of DNA from each sample were pooled and cleaned using the UltraClean PCR Clean-Up Kit (MoBio, Carlsbad, CA). Sequences were generated on three runs using a MiSeq personal sequencer (Illumina, San Diego, CA).

### 2.5 Sequence data analysis

Raw sequences were quality filtered and assigned to samples based on their barcodes using the default parameters of QIIME version 1.5.0 (26). Sequences were assigned to 97% identity operational taxonomical units (OTU)s by comparing them to a nonredundant reference database of near-full length sequences (Greengenes database) (27), and unassigned sequences were clustered into *de novo* OTUs using UCLUST (28). Since samples contained between 4,694 and 72,828 sequences, analyses were standardized at 4,600 sequences per sample to avoid biases. UniFrac (29) PCoA analyses were conducted using QIIME. Bacterial families and genera in each sample were determined using the RDP classifier retrained on the Greengenes taxonomy (30).

### 2.6 Fecal sIF and microbiome PCoA analysis

Fecal sIF data was normalized by dividing each individual value by the mean value of that sIF across all samples. All data were entered into a feature table with 17 features to perform analysis using QIIME2 (31). Distances between fecal samples based on their sIF levels were calculated using Canberra distances. PCoA ordination and biplot functionality in QIIME2 visually integrate the sample feature metadata. Microbiome beta diversity was calculated using unweighted UniFrac and plotted by PCoA. A one-sided Mantel test with Pearson correlation was performed to test for a correlation between the two distance matrices from the microbiome data (unweighted UniFrac) and the immune data (Canberra).

### 2.7 Fecal solute stimulation of intestinal epithelial cells

Human intestinal epithelial T84 cells were grown and maintained in DMEM nutrient mixture F-12 ham (DMEM F-12) media (Gibco, Grand Island, NY) as previously described (32). Cells were plated on permeable transwell inserts (Costar, Cambridge, MA) and grown to confluency and high resistance (>1,000 Ω•cm2). Agonist-stimulated short circuit currents (Isc) were measured in Hank’s balanced salt solution (Sigma-Aldrich) on the apical side using an EVOM2 voltohmmeter (World Precision Instruments, Sarasota, FL). Measurements were taken before fecal solutes were added, 30 minutes post-addition of fecal solutes, and then once an hour for four hours. Cl- secretory responses are expressed as a change in short circuit current (ΔIsc) as previously described (33).

### 2.8 Statistical analysis

Statistical analyses comparing differences between cohorts and correlations for clinical measures, sIF levels, ΔIsc and PC values were performed using GraphPad Prism Version 7 (GraphPad, San Diego, CA). These consist of Kruskal-Wallis with Dunn’s corrections and rank Spearman correlation analyses corrected for multiple comparisons with the false discovery rate (FDR) method of Benjamini and Hochberg (34) were used to determine significance of differences between cohorts. Any correlations where P < 0.05 after FDR correction was considered statistically significant.

## 3 Results

### 3.1 Gastrointestinal symptoms and inflammation are increased in MSM with and without HIV infection

There was no significant difference in the age between cohorts except for the MSM-ART cohort, which was significantly older (**Table 1**). There was also no significant difference in the median CD4^+^ T cell count between participants with HIV with or without ART indicating those in the MSM-HIV cohort were fairly healthy (**Table 1**). To ensure that stool consistency was not responsible for differences in sIFs, we also measured the total protein concentration of the fecal solutes (total BCA) and no significant difference between cohorts was noted (**Table 1**).

**Figure 1:**
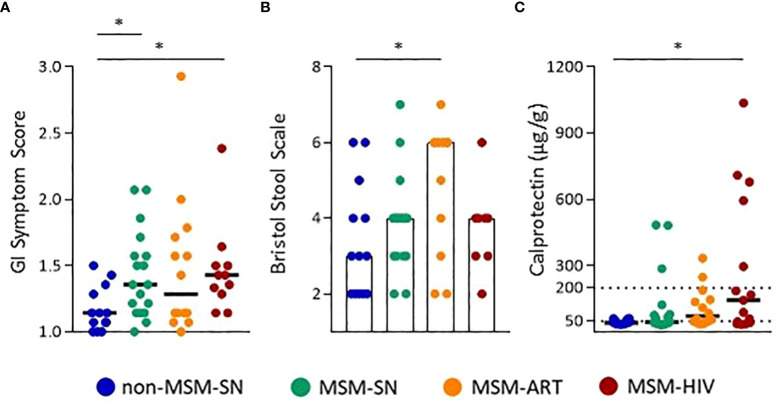
Increased GI symptoms and inflammation in MSM with and without HIV infection. **(A)** GI Symptom Scores, and **(B)** Bristol Stool Scale Scores calculated from survey responses. **(C)** Calprotectin levels (µg/g) determined by ELISA. Each point represents data from one participant and are colored based on cohort: non-MSM-SN (dark blue), MSM-SN (green), MSM-ART (orange) and MSM-HIV (red). Black lines/hollow bars represent the median of each cohort. The dotted line at 200 µg/g represents the cutoff for clinically significant fecal calprotectin. Kruskal-Wallis tests were used to determine statistical significance with Dunn’s multiple comparisons test where * = p < 0.05, ** = p < 0.01, and *** = p < 0.001.

To evaluate differences in overall GI discomfort between our cohorts the gastrointestinal symptom rating survey (GSRS) (24) was completed by all study participants, and aggregate scores compared. Both MSM-SN and MSM-HIV reported a statistically significant increased GI symptom frequency and severity compared to non-MSM-SN (P=0.031; P=0.029, respectively) (**Figure 1A**). After providing a stool sample participants were asked to rate the sample’s consistency using the Bristol stool scale (25), and MSM-ART participants reported more watery/loose stool consistency than non-MSM-SN participants (P=0.039) (**Figure 1B**). We then measured fecal calprotectin, a quantitative clinical marker of GI inflammation. Only MSM-HIV participants had statistically elevated levels of calprotectin in their fecal samples compared to non-MSM-SN (P=0.014) (**Figure 1C**). However, all MSM cohorts had a higher proportion of participants with calprotectin levels over 50 μg/g than the non-MSM-SN cohort (non-MSM-SN: 21.4%, MSM-SN: 44.4%, MSM-ART: 64.3%, MSM-HIV: 66.7%), which can indicate potential GI inflammatory disease (35). Several MSM participants had fecal calprotectin levels greater than 200 μg/g which is strongly associated with GI inflammatory disease (35) (16.7%, 14.3%, and 33.3% of MSM-SN, MSM-ART, and MSM-HIV respectively) while none in the non-MSM-SN cohort had comparable levels. All three of these measures were also examined in F-SN and F-HIV and while the aggregate GI symptom score was significantly higher for F-HIV compared to F-SN (P=0.047) there was no difference in stool consistency (**Supplementary Figure 1A**). Fecal calprotectin showed a similar pattern as for HIV-positive men but was a smaller cohort and did not reach statistical significance (**Supplementary Figure 1A**). Based on both reported symptoms and fecal calprotectin we show a more inflammatory GI environment in HIV-positive individuals and in MSM compared to non-MSM and HIV-SN, which prompted further exploration into the specific characteristics of this inflammation.

### 3.2 Distinct inflammatory MSM fecal sIF profile is exacerbated with HIV infection

**Figure 2:**
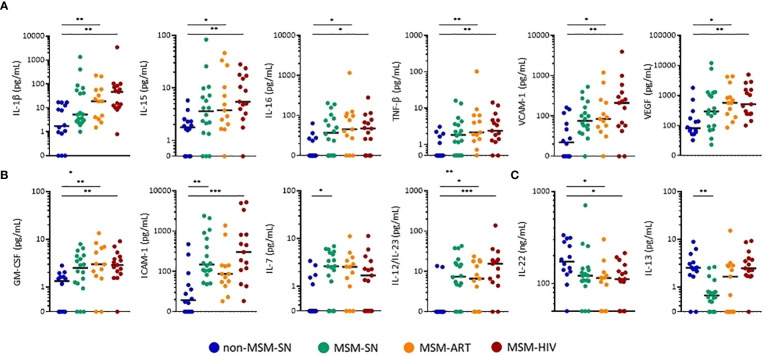
Concentrations of fecal sIFs are altered in MSM compared to non-MSM. Levels of **(A)** IL-1β, IL-15, IL-16, TNF-β, VCAM-1, VEGF (pg/mL), **(B)** GM-CSF, ICAM-1, IL-7, IL-12/23 (pg/mL), **(C)** IL-13 (pg/mL), and IL-22 (ng/mL) comparing MSM-SN, MSM-ART, and MSM-HIV cohorts to non-MSM-SN. Each point represents data from one participant and are colored based on cohort: non-MSM-SN (dark blue), MSM-SN (green), MSM-ART (orange) and MSM-HIV (red). Black lines represent the median of each cohort. Kruskal-Wallis tests were used to determine statistical significance with Dunn’s multiple comparisons test where *p < 0.05, **p < 0.01, and ***p < 0.001.

Twenty-seven sIFs, excluding calprotectin, were measured from fecal samples using both multiplex and standard ELISAs. Seventeen of these were measurable within the standard ranges of each assay for more than 75% of all samples tested. Ten markers (IFN-γ, IL-2, IL-4, IL-6, IL-10, IL-12p70, SSA, IL-5, IL-17A, TNF-α) where less than 75% of participants had detectable levels in their fecal solute were excluded. Of those seventeen, twelve sIFs showed significant differences across cohorts and values for all participants (**Figure 2**). The five sIFs that were detectable but did not have significantly different fecal levels between cohorts were CRP, IL-1α, IL-8, sCD14 and sIgA. Nine (GM-CSF, ICAM-1, IL-1β, IL-12/23, IL-15, IL-16, TNF-β, VCAM-1, and VEGF) were elevated in either cohort of participants with HIV compared to non-MSM-SN (**Figures 2A, B**). The most striking example of fecal sIF elevation for PLWH was seen in IL-1β, where there were 27-fold (MSM-HIV) and 10.9-fold (MSM-ART) increases compared to the non MSM-SN cohort. Many sIFs were highest in MSM-HIV, though still significantly elevated in MSM-ART with the exception of ICAM-1 which was not significantly higher compared to non-MSM-SN. Interestingly, three sIFs (GM-CSF, ICAM-1 and IL-12/23) were also significantly higher in MSM-SN compared to non-MSM-SN and IL-7 was not elevated in either HIV cohort (**Figure 2B**). We also compared non-MSM-SN to MSM-SN using a Mann-Whitney T test. In this analysis, seven (GM-CSF, ICAM-1, IL-7, IL-12/23, IL-16, TNF-β and VCAM-1) of the sIFs were significantly elevated while one (IL-13) was lower in HIV-negative MSM compared to HIV-negative non-MSM (**Supplementary Table 1**). In contrast, IL-22 levels were lower across MSM cohorts compared to non-MSM, most significantly in participants with HIV, and IL-13 was lower in MSM-SN alone (**Figure 2C**). While fecal CRP, IL-1α, IL-8, sCD14 and sIgA levels were detectable for the majority of participants, there were no significant differences between cohorts (data not shown). A separate comparison of only MSM cohorts was done where both MSM-HIV and MSM-ART were compared to MSM-SN and the only significant difference was elevated IL-13 in MSM-HIV (**Supplementary Table 2**). There were no other significant differences between the MSM cohorts. Taken together, our data show significant differences in sIF levels for MSM compared to non-MSM that is present regardless of HIV-infection, and further elevated in participants with HIV.

Levels of fecal sIFs were also examined in the cohorts of female participants. Because we were unable to recruit enough male non-MSM with HIV, we examined a small cohort of females with HIV as their microbiome is similar. Significant increases were observed in F-HIV compared to F-SN for IL-15 (p=0.016), IL-16 (p=0.0079), TNF-β (p=0.0079), GM-CSF (p=0.0079), and IL-12/23 (p=0.0079) (**Supplementary Figure 1B**). Most of these trends align with the observations of fecal sIFs in the male cohorts; however, for IL-1β, ICAM-1 and IL-22 there was no significance between F-HIV and F-SN. Due to the small size of the female cohorts and known gut microbiome differences of MSM (17) the remainder of analyses focused on male participants.

### 3.3 Fecal sIF frequencies correlate with fecal calprotectin

Fecal calprotectin is a standardly used clinical marker for diagnosis and monitoring of inflammatory gut diseases, so we examined associations between fecal calprotectin and sIF levels to better understand their role in GI inflammation. Statistically significant correlations were found between fecal calprotectin levels and IL-1β, IL-8, IL-15, IL-16, GM-CSF, TNF-β, VEGF-A and VCAM-1 (**Table 2**). The most significant of these correlations was between calprotectin and IL-1β (P=0.0005, r=0.51), an inflammatory cytokine associated with GI disease (36) (**Table 2**). In fact, most of the fecal sIFs associated with calprotectin have inflammatory properties (IL-1β, GM-CSF, TNF-β, IL-8, IL-16, and VEGF-A) or are involved in trafficking of leukocytes into tissue (VCAM-1). While the significant correlations between calprotectin and inflammatory sIFs indicate these markers are relevant to overall gut inflammation, it is notable that the two cytokines that were decreased in one or all MSM cohorts, IL-13 and IL-22, did not correlate with calprotectin levels. Additionally, none of the fecal sIFs correlated with the aggregate GSRS or Bristol stool scores. The associations between fecal sIF concentrations and fecal calprotectin indicate sIFs contribute to clinically significant GI inflammation.

**Table 2:** Fecal calprotectin correlates with fecal sIF levels.

Correlate	R-value	P-value
IL-1β	0.5111	0.0005
VEGF	0.4692	0.0017
IL-15	0.4502	0.0017
VCAM-1	0.4384	0.0021
IL-16	0.4258	0.0024
GM-CSF	0.4156	0.0028
IL-8	0.4153	0.0024
TNF-β	0.4127	0.0023
IL-12/23	0.3662	0.0075
IL-7	0.3598	0.008
ICAM-1	0.3365	0.013
IL-1α	0.313	0.021

Significant correlations between fecal sIF concentrations and fecal calprotectin. Test results for ranked Spearman correlations with an FDR<0.05 are shown, followed by the corrected p-value.

### 3.4 Fecal sIF profile is related to microbiome compositional differences

**Figure 3:**
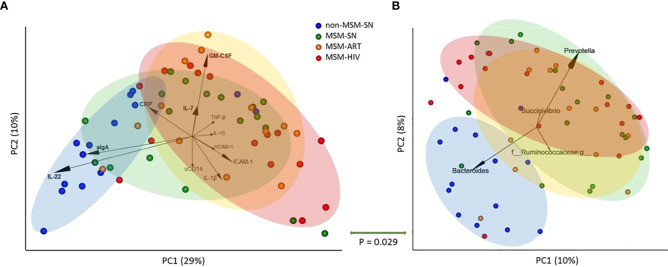
Principal coordinate analyses of fecal sIF and microbiome composition segregate by MSM status. **(A)** PCoA plot of fecal sIF composition after normalization across samples. Each point represents one participant’s sIF composition where the distance between points is representative of relative similarity – two points closer together are more similar than two further apart. Labeled arrows of the 11 most influential sIFs on this PCoA’s distribution are included with corresponding directionality and impact as shown by the arrow’s length. **(B)** Unweighted UniFrac PCoA plot of fecal microbiome composition where each point represents one individual’s overall microbiome composition. Labeled arrows of the 4 most influential taxa on this PCoA’s distribution are included with corresponding directionality and impact as shown by the arrow’s length. Points are colored based on cohort: non-MSM-SN (dark blue), MSM-SN (green), MSM-ART (orange) and MSM-HIV (red). Each colored oval encircles the majority of participants in the cohort with corresponding color. The size and location were determined manually and <20% of each cohort falls outside the oval. The P value below the double-sided green arrow between plots is the result of a Mantel test that shows a significant positive correlation between these two matrices.

Next, we compared the overall fecal sIF profile to microbiome composition. The evaluation of global differences in sIFs was made by performing a principal coordinate analysis (PCoA) of Canberra distances calculated from sIF profiles after values were normalized (**Figure 3A**). Non-MSM-SN individuals clustered separately from MSM cohorts across principle coordinate 1 (PC1), which was separated by elevated IL-22 and sIgA for non-MSM-SN and higher levels of ICAM-1, VCAM-1, IL-1β, TNF-β, and GM-CSF for MSM (**Figure 3A**). Clustering of MSM-ART and MSM-HIV cohorts largely overlapped and were both distinct from non-MSM-SN, while the MSM-SN cohort was more diffuse. We and others have reported that the enteric microbiome of MSM with and without HIV infection is distinctly different than that of non-MSM, in part due to an increase in *Prevotella* and decrease in *Bacteroides (* 17). To relate the microbiome composition to fecal cytokine profiles, we generated 16S rRNA sequence data from the same fecal samples. As shown previously (17), MSM microbiome compositions did not clearly cluster by HIV or ART status using PCoA (**Figure 3B**). Differences in between MSM and non-MSM were again associated with relatively Prevotella rich and Bacteroides poor microbiome composition in the MSM. Comparison of the pairwise distance matrices of the fecal sIF data and unweighted UniFrac values with a mantel test showed a significant relationship (P=0.029) indicating that the microbiome differences explained some of the variation in sIF profiles across fecal samples. We also looked at the abundances of *Prevotella and Bacteroides* individually and found patterns similar to our previous study (17) (**Supplementary Figures 2A–C**). When these abundances were correlated with fecal sIF concentrations *Prevotella* did positively associate with IL-1β levels (P=0.038, r=0.48) and negatively with IL-22 (P=0.004, r=-0.52) for all participants, but there were no correlations within MSM cohorts (**Supplementary Figure 2D**).

### 3.5 Inflammatory sIF composition of MSM with HIV associates with decreased intestinal barrier integrity in colonic epithelial cells

**Figure 4:**
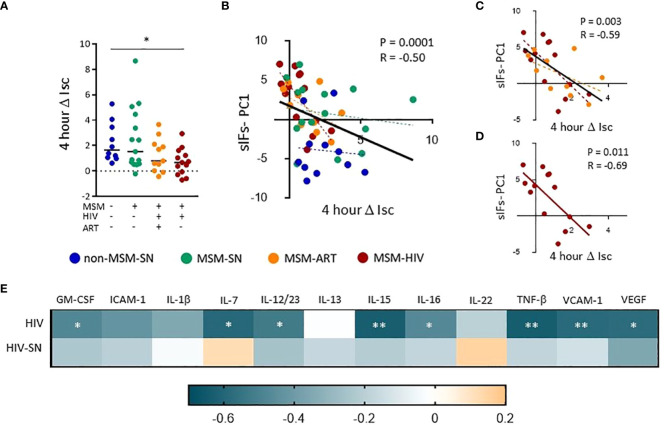
Fecal solutes from MSM with HIV infection increase transcellular gut epithelial permeability and associate with sIFs. **(A)** The change in short circuit current (ΔIsc) after 4 hours was calculated using Ohm’s law. A Kruskal-Wallis test was performed to determine statistical significance where * = p < 0.05, ** = p < 0.01, and *** = p < 0.001. Correlations between ΔIsc and sIF PC1 values from PCoA from figure 3 for all **(B)**, HIV **(C)**, and HIV-ART **(D)** participants. Each point represents data from one participant and are colored based on cohort: non-MSM-SN (dark blue), MSM-SN (green), MSM-ART (orange) and MSM-HIV (red). Each black line represents the linear regression for all included points, and P- and R-values are associated with this line. Each dotted line represents the linear regression for the cohort with the corresponding color. **(E)** A heat map showing associations between sIF concentrations and 4 hour ΔIsc. Teal indicates a negative R-value whereas orange represents a positive R-value. Rank order spearman correlations were run to determine statistical significance where * = p < 0.05, ** = p < 0.01, and *** = p < 0.001.

To assess the effects of sIFs on the colonic epithelial barrier we added fecal solutes to the apical portion of confluent T-84 gut epithelial cells at resistance (1000 Ω•cm2). We measured short circuit current (Isc), a measure of apical fluid transport (37), over a 24-hour period of time and found the peak change from the initial Isc occurred at 4 hours (ΔIsc). We found fecal solute from MSM-HIV induced a significantly lower ΔIsc compared to non-MSM-SN (p = 0.033) and MSM-ART also trended lower (**Figure 4A**). None of the fecal solutes from non-MSM-SN had a negative ΔIsc whereas solutes from 18% and 31% of the MSM-ART and MSM-HIV cohorts respectively decreased Isc compared to the initial reading. We then compared PC1 values of the sIF composition PCoA to ΔIsc using a stratified analysis and found significant associations between PC1 and ΔIsc for all participants (P=0.0003, r=-0.50), participants with HIV (P=0.003, r=-0.59), and MSM-HIV (P=0.011, r=-0.69) while there was no correlations in other stratifications by HIV infection status or by cohort (**Figures 4B–D**). Positive PC1 values, categorized by increased concentration of inflammatory sIFs ICAM-1, VCAM-1, IL-15 IL-1β and TNF-β and decreased IL-22 and sIgA, were associated with decreased gut barrier integrity. There were no correlations found between ΔIsc and microbiome PC1 or levels of individual sIFs for all participants or when stratified by cohort. (**Supplementary Data 1**). We repeated this analysis stratified based on participants’ HIV status and eight of the 12 sIF frequencies significantly correlated with ΔIsc (**Figure 4E**). The strongest associations among participants with HIV involve IL-15 (P=0.0072, r=-0.63), TNF-β (P=0.0096, r=-0.61) and VCAM-1 (P=0.0099, r=-0.59), and GM-CSF, IL-7, IL-12/23, IL-16 and VEGF-A also negatively associate with ΔIsc (**Figure 4E**). There were no significant associations among participants without HIV. These findings indicate the reduced apical fluid transport of the gut epithelial barrier is associated with elevated concentrations of inflammatory sIFs in participants with HIV.

### 3.6 Elevated systemic sCD14 in HIV is associated with fecal sIF induced intestinal barrier dysfunction

**Figure 5:**
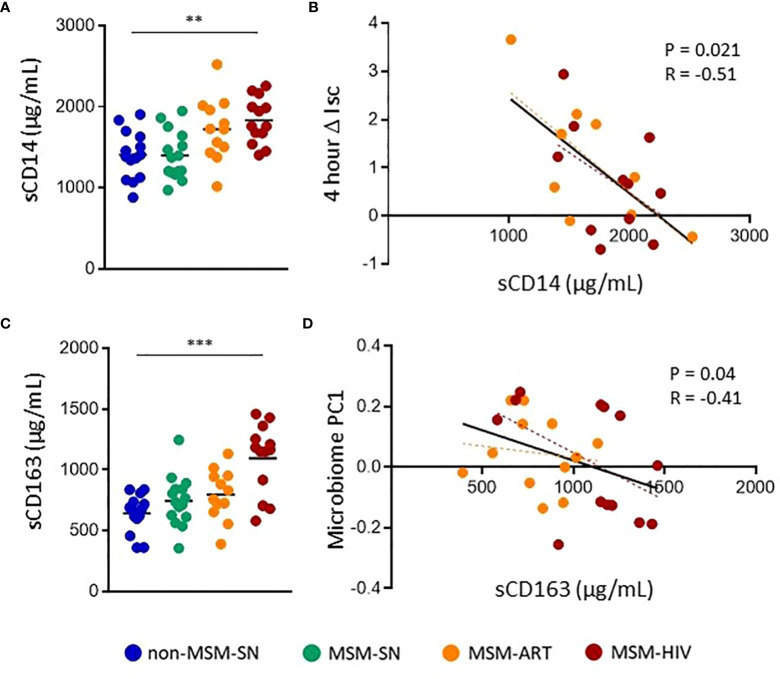
Elevated plasma markers of bacterial translocation in MSM-HIV correlate with increased gut epithelial permeability and microbiome composition. **(A)** Plasma sCD14 levels determined by ELISA and separated by cohort. **(B)** Correlations between ΔIsc and plasma sCD14 for participants with HIV. **(C)** Plasma sCD163 levels determined by ELISA and separated by cohort. **(D)** Correlations between Microbiome PC1 and plasma sCD163 for participants with HIV. For **(A)** and **(C)**, Kruskal-Wallis tests were used to determine statistical significance between cohorts where * = p < 0.05, ** = p < 0.01, and *** = p < 0.001. For **(B)** and **(D)** rank order spearman correlations were run where * = p < 0.05, ** = p < 0.01, and *** = p < 0.001. Each point represents data from one participant and are colored based on cohort: non-MSM-SN (dark blue), MSM-SN (green), MSM-ART (orange) and MSM-HIV (red). Each black line represents the linear regression for all included points and reported P- and R-values are associated with this line. Each dotted line represents the linear regression for the cohort with the corresponding color.

Lastly, to examine the associations between barrier function and systemic inflammation, we measured levels of inflammatory sIFs in plasma. Few significant differences between MSM and non-MSM cohorts in plasma sIFs were noted, and many sIF trends are antithetical to sIFs in the feces (**Supplementary Table 3**). Of note, IL-22 levels in plasma were increased in all MSM cohorts compared to non-MSM, while they were decreased in feces, and of twelve matched sIFs in blood and feces only three positively correlated (**Supplementary Table 3**). We also measured plasma sCD14 and sCD163 to assess the connections between sIF levels and bacterial translocation (38, 39). Plasma sCD14 was significantly elevated in MSM-HIV (P=0.0050, Median=1760 μg/mL) and trended higher in MSM-ART (P=0.074, Median=1726 μg/mL) compared to non-MSM-SN (Median=1398 μg/mL) (**Figure 5A**). There was no significant difference in plasma sCD14 between MSM-SN and non-MSM-SN (P>0.99, Median=1376 μg/mL) (**Figure 5A**). ΔIsc was found to negatively correlate with sCD14 (P=0.018, r=-0.38) for all participants (**Supplementary Figure 3A**) and the relationship was even stronger for participants living with HIV (P=0.021, r=-0.51) (**Figure 5B**). There were no significant associations when stratified by cohort or among HIV-seronegative participants. Additionally, there was a much weaker association between plasma sCD14 and fecal sIF PC1 (P=0.07, r=0.29) and no association with fecal microbiome PC1 (P=0.87, r=0.02) (data not shown). Of the 12 fecal sIFs tested there was one significant association between VEGF and plasma sCD14 (P=0.029, r=0.40) (**Supplementary Table 4**). Plasma sCD163 was also significantly elevated in MSM-HIV (P=0.0003, Median=1166 μg/mL) compared to non-MSM-SN (Median=675 μg/mL), but neither MSM-ART (Median=734 μg/mL) or MSM-SN (Median=794 μg/mL) were significantly higher (**Figure 5C**). While plasma sCD163 did not have significant associations with sIF composition, barrier function or microbiome composition for all participants (**Supplementary Figure 3B**), when stratified by HIV status participants with HIV show a significant correlation between sCD163 and gut microbiome composition (P=0.04, r=-0.41) (**Figure 5D**). There were no significant associations when stratified by cohort. Additionally, there were no significant correlations between individual fecal sIF levels and plasma sCD163 (data not shown). These findings connect systemic inflammation markers to both intestinal barrier function and microbiome composition, both of which are also associated with fecal sIF composition.

## 4 Discussion

HIV infection has long been associated with gastrointestinal disease. Acquired immune deficiency syndrome (AIDS) was initially classified as a chronic wasting disease because of the severe diarrhea and malabsorption seen in PLWH without ART treatment (40). In recent years, profound depletion of CD4^+^ T cells in the intestine and increased bacterial translocation from the gut have been strongly associated with HIV disease progression (41, 42), and these findings have sparked renewed interest of the role of the gastrointestinal tract in HIV pathogenesis. One area of particular interest is the intersection between the gut microbiome and inflammation in HIV, in part due to the strong connections between microbiome composition, inflammatory gut diseases and various chronic conditions (43, 44). Our group and others have shown that HIV infection is associated with intestinal microbiome dysbiosis (45, 46) but interestingly, it has only been recently determined that sexual behavior contributes more significantly to alterations in microbiome composition in HIV-infected MSM than HIV infection itself (17). Understanding the interactions between the gut microbiome, inflammation and how these factors influence HIV pathogenesis and transmission following receptive anal intercourse (RAI) is of interest considering the known associations between vaginal dysbiosis and HIV transmission (47, 48). The relationship between fecal soluble immune factors (sIFs), many of which are important in gut inflammation (36, 49), and the gut microbiome and barrier function has not been previously examined in the context of HIV. Here we show that MSM-SN have higher levels of GI inflammation than non-MSM-SN, and that many of these sIF levels are further elevated in MSM with HIV infection. We also found there was a significant association between the inflammatory composition of fecal sIFs with microbiome composition and associations between individual gut microbiome species and sIF concentrations in a stratified cohort analysis. Lastly, we found both the overall composition of sIFs and increased levels of specific sIFs in MSM with HIV associated with decreased transcellular fluid transport, which in turn strongly associated with measures of bacterial translocation in the plasma of the participants.

We initially assessed gut health and inflammation in our study cohorts using standard clinically validated techniques; GI symptoms questionnaire (24), self-reported Bristol Stool scale (25) and fecal calprotectin (35). As expected, participants with untreated HIV infection reported more GI symptoms compared to non-MSM-SN; however, surprisingly we found that MSM-SN participants also reported more GI symptoms. This could be explained by the higher rate of sexually transmitted infections (STIs) among MSM than non-MSM populations (50), which previously were thought to be the primary cause of increased gut inflammation in MSM. However, there were no significant correlations between sIF levels and either stool consistency or the average severity of reported GI symptoms. Additionally, the rates of STIs – including *Neisseria gonorrhoeae* and *Treponema pallidum* which cause gonorrhea and syphilis, respectively – have increased in recent years among MSM and may be influenced by increased use of pre-exposure prophylaxis for HIV and associated behavioral changes (51); however, these bacteria were not detected in our microbiome analysis and no viral or parasitic STIs were reported by participants. Another notable finding was that the stool from MSM-ART participants had a looser consistency compared to non-MSM-SN. This aligns with known associations between diarrhea and other GI symptoms with various ART regimens (52). We also measured the levels of fecal calprotectin which is directly related gut inflammation (53). HIV-ART participants were more likely to have elevated levels of gut inflammation than non-MSM-SN, marked by fecal calprotectin between 50μg/g and 200μg/g (35), and fecal calprotectin in ART-naïve participants was significantly higher compared to non-MSM-SN. In fact, many had levels typical of those observed with IBD which is similar to previous findings in HIV infection (54). We were surprised to see that 44% of MSM-SN had fecal calprotectin above 50 μg/g while only 21% of non-MSM-SN participants had comparable levels. Cumulatively, these findings further solidify the connection between MSM, regardless of HIV infection, and GI inflammation and prompted further exploration into how a broad range of fecal sIFs differ with both HIV infection and MSM status.

Analysis of stool sIFs has been previously used to study various inflammatory diseases in the gastrointestinal tract (20–23). These studies found that analysis of fecal sIFs provide a sensitive and noninvasive measurement of gastrointestinal inflammation and provide information on the intestinal environment during or after viral infection (55). Of the 17 sIFs that fell into range, eleven were elevated in MSM with HIV compared to seronegative non-MSM and many of these are associated with inflammatory GI diseases. Both MSM-HIV and MSM-ART had elevated levels of eight inflammatory sIFs (GM-CSF, IL-1β, IL-12/23, IL-15, IL-16, TNF-β, VCAM-1 and VEGF), while ICAM-1 was increased in MSM-HIV compared to non-MSM-SN. Of these, IL-1β, IL-12/23, IL-16 and TNF-β are all elevated in IBD and associated with its pathogenesis (36, 56, 57), IL-15 and GM-CSF are elevated in inflamed intestinal tissues (58, 59), and anti-VEGF therapies have been shown to reduce intestinal inflammation in models of IBD (60). Recently elevated IL-23 in the gut has also been associated with disease severity after infection with SARS-CoV-2, which has a high incidence of GI symptoms (55). Since these sIFs have strong connections to inflammatory diseases and viral infections, it is unsurprising to see elevated fecal levels in PLWH but levels of four sIFs (GM-CSF, ICAM-1, IL-7 and IL-12/23) were similarly elevated in MSM-SN compared to non-MSM-SN. Both GM-CSF and IL-12/23 are pro-inflammatory, while ICAM-1 and IL-7 modulate the trafficking of immune cells into tissues and proliferation of lymphocytes respectively, all functions that support epithelial barrier integrity (59, 61–63). The elevation of these sIFs in MSM-SN is reasonable following increased prevalence of mechanical injuries and pathogenic microbes in the rectum and colon, both of which can negatively affect gut barrier health. Additionally, IL-16, TNF-β and VCAM-1 were also significantly higher in MSM-SN when directly compared to non-MSM-SN indicating GI inflammation, while exacerbated by HIV infection, is elevated in seronegative MSM. Decreased IL-22 was observed across MSM cohorts, most significantly in participants with HIV. This may be functionally significant since IL-22 – produced in the gut by CD4^+^ T helper subsets and innate lymphoid cells (ILCs) – is known to promote epithelial barrier integrity (64–66). This is particularly important in the context of HIV since IL-22 producing CD4^+^ T cells are preferentially infected by HIV and both these T cells and ILCs are significantly depleted in the gut even following long-term ART (66–68). Surprisingly, of the twelve sIFs that were detectable in both the blood and feces of these participants only three – CRP, IL-16 and VCAM-1 – had significant direct correlations. Interestingly, while IL-22 was lower in both HIV cohorts compared to non-MSM-SN in feces, these levels were significantly elevated in all MSM cohorts in the blood. While twelve of the seventeen sIF levels positively associated with fecal calprotectin, there were five sIFs, including IL-22, that did not have significant associations. Taken together, this indicates that fecal sIFs measurement reveals immune processes that are specific to the gut, are a valid measurement of inflammation and more sensitive than fecal calprotectin alone. These data provide broader insight into intestinal inflammation than other non-invasive measures and allow for a deeper understanding of the different interactions of the gut microenvironment of MSM.

Evaluation of the differences of fecal sIF profile by PCoA showed clustering of MSM and non-MSM cohorts. This analysis indicated IL-22 levels contributed the most of any sIF to the clustering observed, and in addition to its contribution to epithelial barrier integrity, IL-22 can modulate gut microbiome composition (69, 70). Particularly commensal *Clostridia*, through the production of short-chain fatty acids (71), can increase IL-22 production in both *in vitro* and *in vivo* models (72). Based on the known differences in microbiome composition in MSM and non-MSM (17) and the microbiome’s connection to IL-22, we hypothesized that the gut microbiome may contribute to elevated inflammatory sIFs. To assess this, we performed a mantel test to compare pairwise distances between fecal sIF profiles and gut microbiome composition. However, the association was weak indicating that other factors that we did not measure, such as sexual practices (73, 74), may also contribute to the differences. We also found plasma sCD163 was elevated in MSM-HIV and associated with gut microbiome composition. A marker of macrophage activation and bacterial translocation (39), sCD163 has also been connected to mortality and viral replication in PLWH (75, 76). Recent studies evaluating the microbiome in MSM with HIV infection have also found both altered microbiome and elevated sCD163 in this population, but the cause of this association remains unclear (77). Interestingly, this relationship between sCD163 and microbiome composition is specific to PLWH, indicating the possibility of a mechanism specific to HIV infection. Based on these findings, further investigation of the relationship between the gut microbiome and sIFs in MSM and PLWH is warranted.

Systemic immune activation in HIV is caused in part by translocation of microbial products from the gut (78, 79). To better understand the effect of sIFs on gut barrier integrity we cultured T-84 cells with total fecal solutes and measured ΔIsc – a measure of apical fluid transport. We found that addition of fecal solutes from MSM-HIV to T-84 monolayers significantly lowered levels of apical fluid transport as compared to non-MSM-SN, while MSM-ART trended similarly. Increased apical fluid transport is associated both with improved mucosal hydration and decreased bacterial translocation (37), therefore fecal inflammatory sIFs could contribute to increased bacterial translocation and decreased barrier integrity associated with HIV (78). These data indicate there is a connection between fecal sIF composition, decreased intestinal barrier function and bacterial induced systemic inflammation. While the fecal solutes used also included bacterial metabolites that are thought to contribute to intestinal barrier function (80), we also found negative associations between apical fluid transport and IL-15, TNF-β, VCAM-1, VEGF, GM-CSF, IL-7, IL-12/23 and IL-13 in participants with HIV. Interestingly, no significant association between sIF concentration and resistance to intracellular transport was seen in participants without HIV, suggesting HIV-specific fecal sIF compositions are more disruptive to the gut barrier. Various cytokines in the intestinal lumen, including IL-12, negatively impact intestinal membrane permeability (81) and IL-15 is known to drive epithelial tissue destruction (82), cause inflammation downstream of the antiapoptotic signals it initiates (83) and direct intraepithelial lymphocyte motility in the gut (84). VEGF overexpression can be pathogen-induced and also contributes to barrier permeability (85). T helper subsets, which are preferentially depleted in the gut of PLWH (86), associate with worse outcomes from infections and are also associated with increased GM-CSF (87). Most importantly, we discovered a significant inverse association between our *in vitro* measure of gut permeability and plasma sCD14 (38), a marker of bacterial translocation levels, indicating that disruption of barrier function *in vitro* correlated with *in vivo* levels of bacterial translocation. As plasma sCD14 is associated with mortality in PLWH even with ART (88) and the mechanisms behind microbial translocation in HIV are not completely understood (89), further investigation into the interactions between sIFs and gut barrier function could reveal new therapeutic targets to decrease systemic inflammation and associated co-morbidities (2, 90) in PLWH. Taken together, these data outline the interactions between fecal sIFs, gut barrier function and bacterial translocation and connects our *in vitro* findings to direct *ex vivo* measurements.

Analysis of fecal sIF allows for a deeper understanding of the gut microenvironment not detectable by other low-risk, noninvasive methods. The relevance of fecal sIF analysis is confirmed by strong relationships with fecal calprotectin, while also illuminating unique mechanistic insights into gastrointestinal inflammation in HIV infection. Here we connect fecal sIFs to the overall composition of the gut microbiome and individual bacterial taxa abundance, barrier function and bacterial translocation. More work is needed to determine if microbiome dysbiosis is causal, or if inflammatory conditions allow for increased abundance of opportunistic bacteria. However, this study clearly shows elevated levels of multiple sIFs in the stool of MSM with and without HIV infection. Furthermore, we show relationships between plasma markers of bacterial translocation and fecal sIF and microbiome compositions further supporting the theory that gut dysbiosis contributes to chronic systemic inflammation in HIV.

## Data availability statement

The microbiome data presented in the study are deposited in the European Nucleotide Archive repository, accession number PRJEB28485. Additional data may be requested through the University of Colorado Institutional Data Access Committee (contact Crao_Contracts@ucdenver.edu) for researchers who meet the criteria for access to confidential data.

## Ethics statement

The studies involving human participants were reviewed and approved by Colorado Multiple Institution Review Board (COMIRB# 14-1595). The patients/participants provided their written informed consent to participate in this study.

## Author contributions

JMS, SC, and BP designed research. JMS, CN, IC, NN, and NM-H performed research. KL, JMS, VS, JCS, AA, CL, and BP analyzed data. KL, JMS, CN, CL, and BP wrote the manuscript. All authors approved the final version of this manuscript to be published.
