# Supplementary material for: Elevated inflammatory fecal immune factors in men who have sex with men with HIV associate with microbiome composition and gut barrier function
Source: Front Immunol. 2022 Dec 20;13:1072720. doi: 10.3389/fimmu.2022.1072720 (PMC9808389; doi:10.3389/fimmu.2022.1072720)
Supplement: Supplementary file 1 [file DataSheet_1.pdf]

Supplementary Material

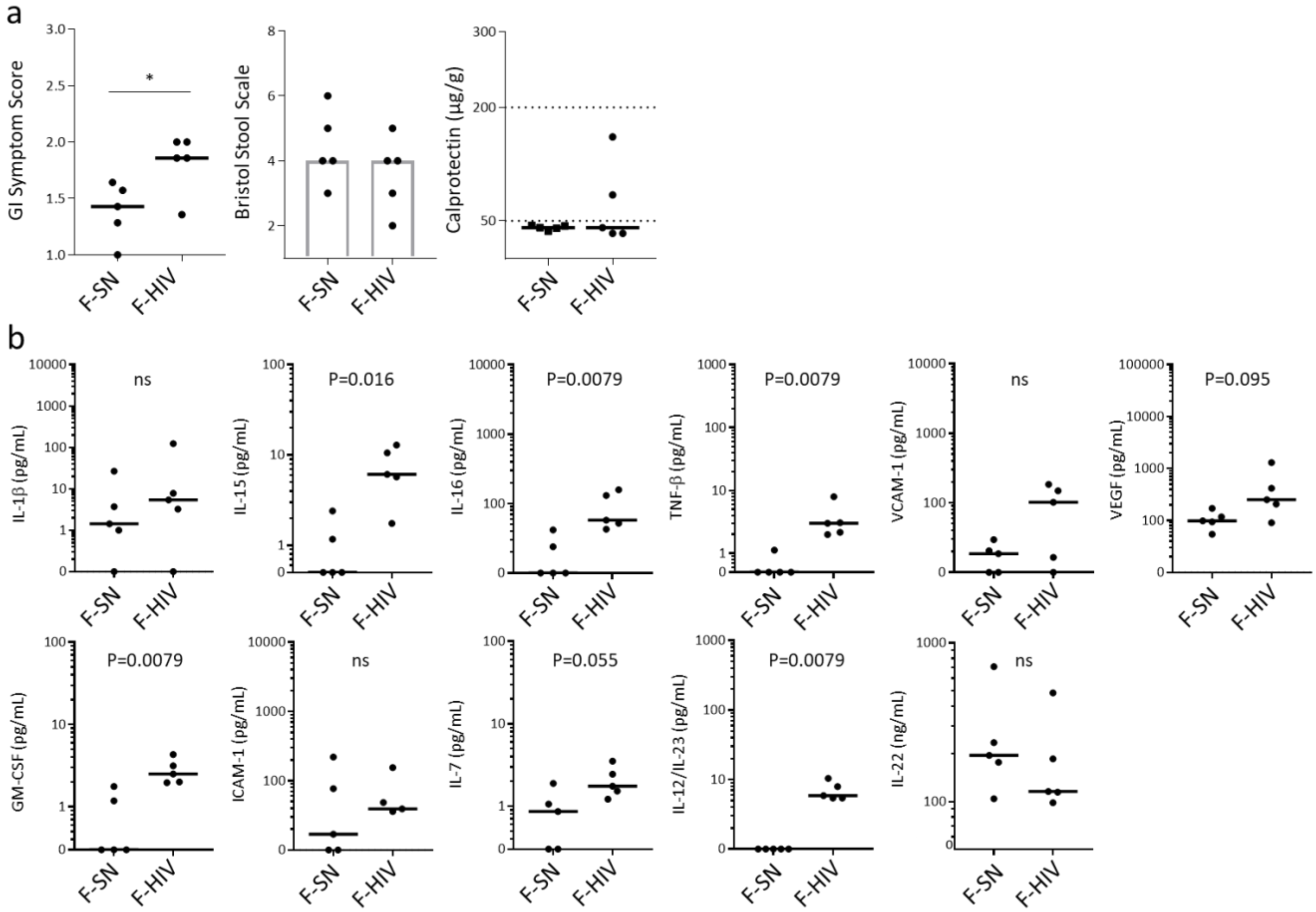

Supplementary Figure 1. GI Inflammation and sIF levels in female participants

(a) GI symptom scores and Bristol stool scale scores calculated from survey responses and calprotectin levels (µg/g) determined by ELISA for female participants. (b) Levels of IL-1β, IL-15, IL-16, TNF- β, VCAM-1, VEGF, GM-CSF, ICAM-1, IL-7, IL-12/23 (pg/mL), and IL-22 (ng/mL) comparing F-SN and F-HIV. Each point represents data from one participant and lines/hollow bars represent the median of each cohort. The dotted lines at 50 and 200 µg/g represent cutoffs for clinically significant fecal calprotectin levels. Mann-Whitney tests were used to determine statistical significance where * = $p < 0.05$, ** = $p < 0.01$, and *** = $p < 0.001$.

Supplementary Table 1. Fecal sIF comparison comparing seronegative cohorts

P-value results of fecal sIF values comparing MSM-SN to non-MSM-SN. Mann-Whitney tests were performed where P values less than 0.05 are underlined and indicate a significant difference compared to the non-MSM-SN cohort.

	MSM-SN
GM-CSF	0.003
ICAM-1	0.021
IL-1β	0.167
IL-7	0.0007
IL-12/23	0.003
IL-13	0.004
IL-15	0.103
IL-16	0.011
IL-22	0.119
TNF-β	0.023
VCAM-1	0.010
VEGF	0.105

Supplementary Table 2. Fecal sIF comparison between MSM cohorts

P-value results of fecal sIF values comparing the MSM-ART and MSM-HIV cohorts to the MSM-SN cohort. Kruskal-Wallis tests with multiple comparisons were performed where P values less than 0.05 are underlined and indicate a significant difference compared to the MSM-SN cohort.

	MSM-ART	MSM-HIV
GM-CSF	0.76	0.74
ICAM-1	0.13	1
IL-1β	1	0.14
IL-7	1	0.45
IL-12/23	1	0.71
IL-13	0.25	<u>0.0009</u>
IL-15	1	0.27
IL-16	1	1
IL-22	0.91	1
TNF-β	0.62	0.62
VCAM-1	0.72	0.18
VEGF	1	0.45

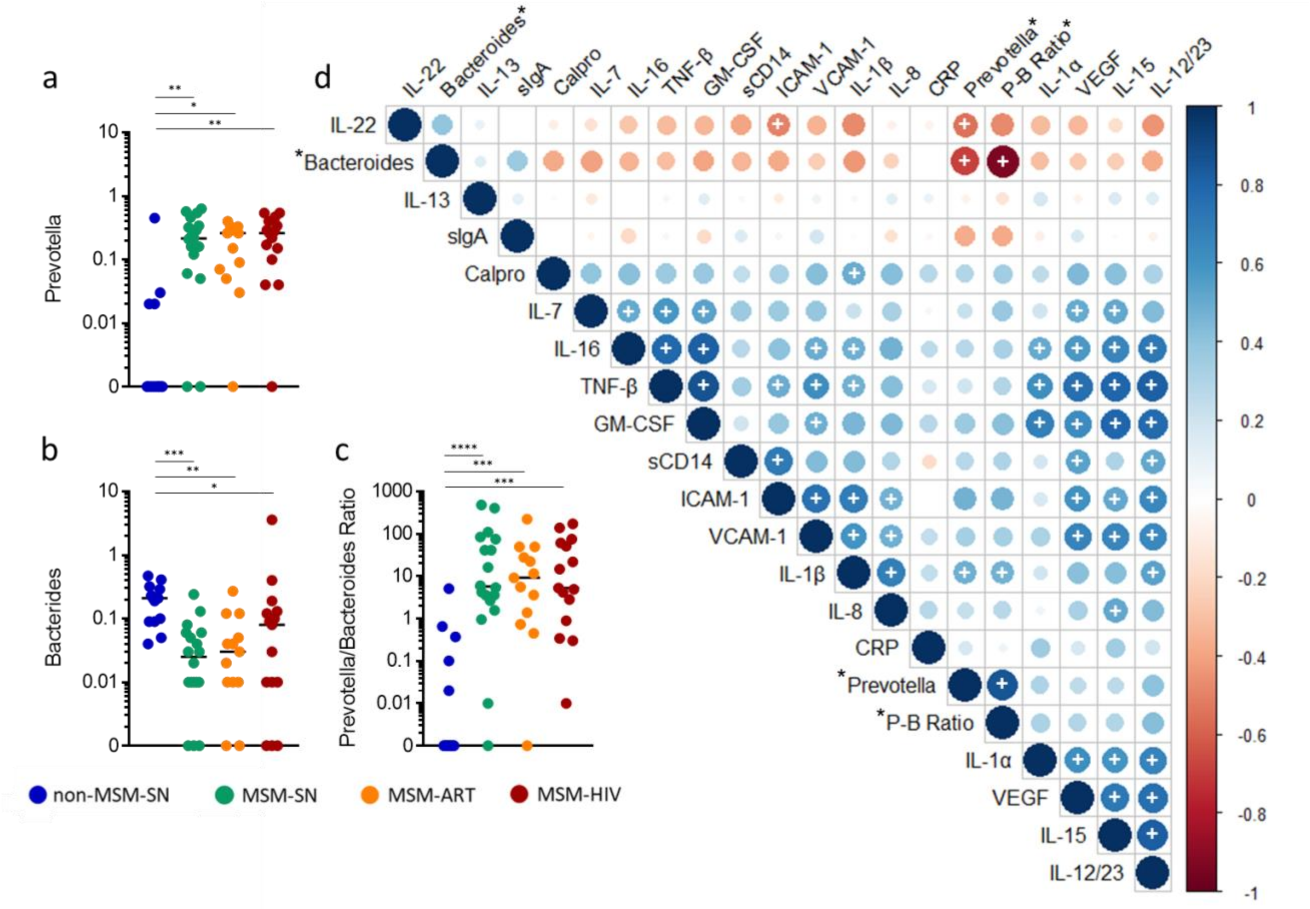

Supplementary Figure 2. Relative abundance of *Prevotella* is higher in MSM compared to non-MSM and correlates with fecal IL-22 and IL-1β

Sum of the relative abundance of all OTUs identified as (a) *Prevotella* and (b) *Bacteroides* genus and (c) the ratio of *Prevotella* frequency by *Bacteroides* frequency comparing MSM-SN, MSM-ART, and MSM-HIV cohorts to non-MSM-SN. Each point represents data from one participant and are colored based on cohort: non-MSM-SN (dark blue), MSM-SN (green), MSM-ART (orange) and MSM-HIV (red). Lines represent the median for each cohort. Kruskal-Wallis tests were performed to determine statistical significance where * = $p < 0.05$, ** = $p < 0.01$, and *** = $p < 0.001$. (d) A correlation matrix of fecal sIFs, *Bacteroides* and *Prevotella* relative abundances and the ratio of *Prevotella* over *Bacteroides* (P-B Ratio). The larger the circle the stronger the R-value and red indicates a negative R-value whereas blue represents a positive R-value. Rank order spearman correlations were run to determine statistical significance where FDR < 0.05 and significant correlations are indicated by a white "+" symbol.

Supplementary Table 3: Average plasma concentration of sIFs for each cohort

Plasma concentrations reported as the median of results for all participants in a cohort. All results are reported as pg/ml except where indicated. Kruskal-Wallis ANOVAs with correction for multiple comparisons were run to determine statistical significance of MSM cohorts compared to non-MSM-SN where * = $p < 0.05$, ** = $p < 0.01$, *** = $p < 0.001$, and Δ = (ng/ml). Plasma values were also compared to corresponding fecal sIF concentrations by Spearman test and † indicates a significant ($p < 0.05$) result.

	non-MSM-SN	MSM-SN	MSM-ART	MSM-HIV
CRP†	1465	1329	4297	2195
GM-CSF	0.25	0.22	0.34	0.43
ICAM-1	343.1	332.0	461.6**	589.8**
IL-7	12.4	15.0	12.6	14.7
IL-12/23	177.2	153.6	209.8	425.1**
IL-15	3.9	3.8	4.2	3.9
IL-16†	394.1	398.1	543.7	440.0
IL-22$^{\Delta}$	225.7	337.3*	348.6*	425.5***
sCD14$^{\Delta}$	1398	1376	1722	1760**
sCD163	675	734	794	1166***
TNF-β	0.22	0.19	0.23	0.40
VCAM-1†	479.7	442.3	696.5**	916.7***
VEGF	63.1	70.0	90.7	113.7

Supplementary Table 4. Fecal VEGF positively associates with plasma sCD14

Significant correlations between fecal sIF concentrations and plasma sCD14. Test results for ranked Spearman correlations with an FDR<0.05 are shown, followed by the corrected p-value.

Correlate	R-value	P-value
VEGF	0.40	0.029
VCAM-1	0.35	0.053
IL-7	0.28	0.16
IL-1β	0.22	0.17
ICAM-1	0.23	0.19
TNF-β	0.25	0.19
IL-15	0.23	0.22
GM-CSF	0.17	0.33
IL-12/23	0.15	0.38
IL-16	0.075	0.71
IL-22	-0.030	0.91
IL-13	0.001	0.99

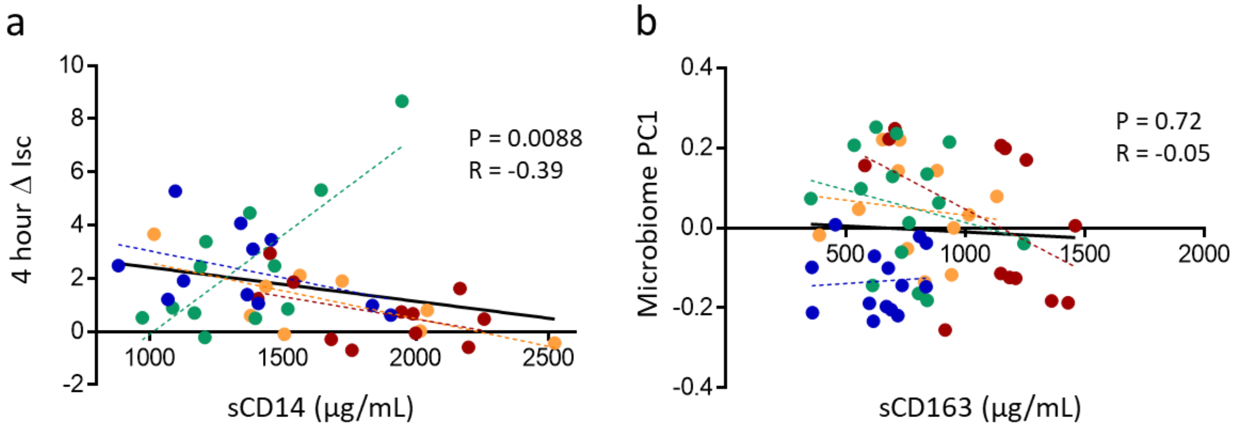

Supplementary Figure 3. Systemic marker correlations with gut epithelial barrier integrity and microbiome composition for all participants

(a) Plasma sCD14 levels determined by ELISA correlated with ΔIsc for all participants. (b) Plasma sCD163 levels determined by ELISA correlated with Microbiome PC1 for all participants. Rank order spearman correlations were run where * = $p < 0.05$, ** = $p < 0.01$, and *** = $p < 0.001$. Each point represents data from one participant and are colored based on cohort: non-MSM-SN (dark blue), MSM-SN (green), MSM-ART (orange) and MSM-HIV (red). Each black line represents the linear regression for all included points and reported P- and R-values are associated with this line. Each dotted line represents the linear regression for the cohort with the corresponding color.
